# Expression Pattern of Seminal Plasma Extracellular Vesicle Small RNAs in Boar Semen

**DOI:** 10.3389/fvets.2020.585276

**Published:** 2020-11-11

**Authors:** Zhiqian Xu, Yanshe Xie, Chen Zhou, Qun Hu, Ting Gu, Jie Yang, Enqin Zheng, Sixiu Huang, Zheng Xu, Gengyuan Cai, Dewu Liu, Zhenfang Wu, Linjun Hong

**Affiliations:** ^1^National Engineering Research Center for Breeding Swine Industry, Guangdong Provincial Key Laboratory of Agro-Animal Genomics and Molecular Breeding, College of Animal Science, South China Agricultural University, Guangzhou, China; ^2^Lingnan Guangdong Laboratory of Modern Agriculture, Guangzhou, China

**Keywords:** seminal plasma, extracellular vesicles, miRNA, piRNA, boar

## Abstract

Extracellular vesicles (EVs) regulate multiple physiological processes. Seminal plasma contains numerous EVs that may deliver functional molecules such as small RNAs (sRNAs) to the sperm. However, the RNA profiles in the boar seminal plasma extracellular vesicles (SP-EVs) and its function have not been characterized. The aim of this study was to characterize the functions and sRNA profiles in the boar SP-EVs using deep sequencing technology. Briefly, boar SP-EVs were isolated by differential ultracentrifugation and confirmed with a transmission electron microscope (TEM), nanoparticle tracking analysis (NTA), and Western blot. The isolated boar SP-EVs contained numerous and diverse sRNA families, including microRNAs (miRNAs, 9.45% of the total reads), PIWI-interacting RNAs (piRNAs, 15.25% of the total reads), messenger RNA fragments (mRNA, 25.30% of the total reads), and tRNA-derived small RNAs (tsRNA, 0.01% of the total reads). A total of 288 known miRNAs, 37 novel miRNA, and 19,749 piRNAs were identified in boar SP-EVs. The identified ssc-miR-21-5p may confer negative effects on sperm fertility based on a dual-luciferase reporter experiment. This study therefore provides an effective method to isolate SP-EVs and characterizes the sRNA profile.

## Introduction

Extracellular vesicles (EVs) are lipid bilayer-delimited spherical structures released by cells into their surrounding microenvironment (1). EVs were first observed during the physiological release of non-specific cell vesicles by reticulocytes as they mature into erythrocytes and are thought to be a mechanism of discarding cell membrane proteins (2). However, EVs are currently understood to be secreted by various cell types (3). EVs fall into two main categories, exosomes, and microvesicles. Exosomes have diameters ranging between 50 and 150 nm, while microvesicles are 50–1,000 nm in diameter (1). Once released, EVs can reach distant sites and transfer their cargoes to target cells, inducing phenotypic changes. Thus, EVs may mediate intercellular communication upon release into extracellular space (4).

Spermatogenesis and post-testicular sperm maturation, capacitation, and acrosome reaction are complex biological processes that need communication between cells and organs (5). For example, changes in sperm morphology and post-testicular sperm function rely on sperm interaction with the intraluminal fluid, especially in the epididymis (6), suggesting that EVs in male reproductive biofluids may participate in intercellular communication before and after spermatogenesis. It has been shown that EVs in seminal plasma may enhance ejaculated sperm motility and influence multiple other biological functions when they fuse to sperm membrane in human (7). Furthermore, it has been reported that boar seminal plasma EVs (SP-EVs) could maintain sperm function by fusing to the sperm membrane (8), indicating that some regulatory materials in the boar SP-EVs, such as small RNAs (sRNAs), may participate in post-testicular sperm function following transfer from EVs to sperms.

Micro-RNAs (miRNAs) are single-stranded RNA molecules composed of 22–24 nucleotides. They are important modulators of post-transcriptional gene expression in mammals (9, 10). MiRNAs exist in testis, sperms, seminal plasma, and SP-EVs, and may play important roles in spermatogenesis, sperm maturation, and capacitation (11). PIWI-interacting RNAs (PiRNAs), which consist of 24–31 nt, are another class of sRNA that could bind to PIWI proteins and modulate germ cell processes, including spermatogenesis (12, 13). PiRNAs are thought to modulate spermatogenesis by targeting specific genes and give novel insights into causes of male infertility (14). However, piRNAs' function in boars has not been thoroughly investigated, especially in boar SP-EVs. Additionally, previous studies showed that human SP-EVs carry various kinds of small non-coding RNAs, including miRNAs, piRNAs, tsRNA, and Y RNA, which have regulatory functions (15, 16). In mice and bovine systems, EVs from different regions of the male reproductive tract have distinct miRNA profiles, which have different biological functions depending on their origin (17, 18). Thus, by directly transferring their sRNA cargoes to target cells, EVs may play important roles in intercellular communication.

Pigs are not only economically important farm animals but also large animal models widely used in biomedical research due to their anatomical and physiological closeness to humans (19, 20). However, few studies have investigated the biological function of boar SP-EVs. Here, we aimed to isolate boar SP-EVs and elucidate sRNA profiles in boar SP-EVs by deep sequencing, thereby providing comprehensive new information of the physiological functions of boar SP-EVs.

## Materials and Methods

### Ethics Statement

Procedures involving boars and semen samples were performed in adherence to guidelines by the Institutional Animal Care and Use Committee (IACUC) of South China Agricultural University.

### Sample Collection

Six healthy, fertile Duroc boars were used as semen donors. The boars were housed in a farm (Yunfu, Guangdong Province, China) under controlled environmental conditions with a temperature between 20 and 22°C and a relative humidity of 60%. The sperm-rich fraction of the ejaculates were collected using the gloved-hand method. All semen samples met the following criteria: >2 × 10^8^ spermatozoa/mL, with at least 70% of spermatozoa exhibiting normal motility and 80% of them morphologically normal. Sperm motility was determined under a light microscope (400×) as the percentage of linear motility at 37°C. Sperm morphological assessment was determined under light microscopy (400×); sperms with a proximal droplet, distal droplet, and head/tail abnormality were considered abnormal. At least 200 spermatozoa were counted per slide.

### Isolation of EVs From Boar Seminal Plasma

EVs were isolated from seminal plasma by differential centrifugation as described previously with some modification (8, 21). Briefly, about 200 mL of semen was centrifuged at 800 *g* for 20 min and then at 2,000 *g* for 20 min at room temperature so as to remove sperms. The supernatant was then centrifuged at 16,000 *g* for 1 h at 4°C to remove residual sperms and cell debris, and the resulting supernatant has been considered as seminal plasma fraction. The seminal plasma fraction was then ultracentrifuged at 120,000 *g* for 1 h at 4°C to obtain the EVs and the EV-depleted seminal plasma fraction. The EV pellets were then rinsed twice with PBS by ultracentrifugation at 120,000 *g* for 1 h at 4°C. Finally, the pellets were resuspended in PBS.

### SP-EVs Characterization

Boar SP-EV morphology was determined by a transmission electron microscope (TEM) with a modification of the protocol as described by Rodriguez et al. (22). Briefly, 10 μL of SP-EVs was loaded onto Cu grids and incubated for 10 min at room temperature. They were then stained with 2% uranyl acetate (aqueous) for 2 min before air drying and examination by TEM (Talos L120C, Thermo Fisher). The sizes of the SP-EVs were analyzed by nanoparticle tracking analysis (NTA) using a Zetaview (Particle Metrix) with a 488-nm laser (23). SP-EVs samples were diluted in PBS for 50 times before NTA, and then analyzed according to manufacturer instructions.

### Western Blot Analysis

SP-EVs were examined for specific markers using Western blot (24). Briefly, sample lysis was done using RIPA buffer supplemented with 1% protease inhibitor cocktail (Beyotime, Cat. No. P0013B). Total protein was quantified using the Rapid Gold BCA protein assay kit (Thermo Fisher, Cat. No. A53225). A total of 20 μg of the protein was denatured and subjected to 12% SDS-PAGE. Proteins were then transferred onto PVDF membranes (Millipore, Cat. No. IPVH08110), blocked with 5% (w/v) skim milk for 2.5 h at room temperature, and washed five times with TBST. The membranes were then incubated with anti-CD9 (Abcam, Cat. No. ab223052, 1:1,000) and CD63 (Abcam, Cat. No. ab216130, 1:1,000) overnight at 4°C. They were then washed five times with TBST, 5 min each time, and incubated with a HRP-conjugated goat anti-rabbit IgG secondary antibody for 2 h at room temperature. Signal was then developed using enhanced chemiluminescence substrate (Beyotime, Cat. No. P0018S) and examined using a UVP system (Upland).

### Total RNA Isolation and sRNA Sequencing

SP-EVs were isolated from 200 mL of seminal plasma, and total RNA was isolated from SP-EVs using exoRNeasy Serum/Plasma Maxi Kit (Qiagen, Cat. No. 77023) according to the manufacturer's instructions. RNA samples were digested with RNase-free DNase I (EN0521, Thermo Scientific). RNA yield and quality were determined using NanoPhotometer® (IMPLEN) and Agilent 2100 pic600 (Agilent Technologies). A total of 3 μg of RNA isolated from six healthy boar SP-EVs were pooled together for RNA library construction. Sequencing libraries were produced by NEBNext® Multiplex Small RNA Library Prep Set for Illumina® (NEB) according to the manufacturer's instructions. Sequencing was done on an Illumina Hiseq 2500/2000 platform, and 50-bp single-end reads were generated.

### Analysis of sRNA Sequence Data

First, clean data (clean reads) from raw data were processed with a customized perl and python scripts. Clean reads were then mapped to the *Sus scrofa* Ensemble 94 genome version (25) using Bowtie (26). To make every unique sRNA mapped to only one annotation, each unique sRNA was annotated by following the priority rule: known miRNA > rRNA > tRNA > snRNA > snoRNA > repeat > gene > NAT-siRNA > gene > ta-siRNA > piRNA > novel miRNA, using miRBase release 22 (27) and RepeatMasker (http://www.repeatmasker.org/), Rfam database (http://rfam.xfam.org/), and the reference pig genome as reference. Next, novel miRNAs were identified according to the precursor hairpin structure and the secondary structure predicted using miREvo (28) and mirdeep2 (29). MiRNA expression levels were calculated by TPM (transcript per million) (30): Normalized expression = mapped read count/Total reads^*^1,000,000. Furthermore, other tags were performed on a transcript database (http://www.ensembl.org) (25) and specific piRNA database piRNABank (http://pirnabank.ibab.ac.in/) (31).

### Quantitative PCR (q-PCR)

Six boar SP-EVs miRNAs were randomly selected (four higher sequence reads miRNAs: ssc-miR-21-5p, ssc-miR-148a-3p, ssc-miR-10a-5p, and ssc-miR-125b, and two lower sequence reads miRNAs: ssc-miR-135 and ssc-miR-744). Reverse transcription was performed according to the TransScript miRNA First-Strand cDNA Synthesis SuperMix kit (Transgen, Cat. No. AT351-01). These cDNAs were validated by SYBR-Green qPCR using PerfectStartTM Green qPCR SuperMix kit (Transgen, Cat. No. AQ602-21) on a Real-Time PCR System (Applied Biosystems). The spike-in control cel-miR 39-1 (Qiagen, Cat. No. 219610) was used as endogenous control. Universal reverse primer for qPCR was obtained from TransScript miRNA First-Strand cDNA Synthesis SuperMix kit (Transgen, Cat. No. AT351-01). All measurements were analyzed in another six boar SP-EVs samples (*n* = 6). Relative miRNA expression was calculated using the 2^−−ΔΔCt^ method (32).

### Prediction of the Target Genes

The target genes of miRNA were predicted by miRanda (33) with score cutoff ≥140 and energy cutoff ≤ −10 kcal/mol, and we use pig genome as a background. Analysis of ssc-miR-21-5p, the most abundant miRNA in the SP-EVs, identified its putative target gene pig *VCL* (Gene ID: 396974), among other genes. This gene is known to play an important role in sperm capacitation. Previous studies have shown that *VCL* is part of the focal adhesion protein complex in the acrosome region and affects sperm capacitation (34).

### Plasmid Construction and Dual-Luciferase Reporter Assay

The normal and mutant of the 3'UTR (untranslated region) of the pig *VCL* gene (Gene ID: 396974) were amplified by PCR and inserted into the pGL3-basic vector (Promega) with Xbal digestion (the sequences refer to Supplementary Data Sheet 1). The PK-15 cells were cultured with Dulbecco's minimum essential medium (DMEM, Gibco) containing 10% fetal bovine serum (Gibco) at 37°C in a humidified 5% CO_2_ atmosphere. When the PK15 cells grew to about 75% confluent, ssc-miR-21-5p (100 nM) and a luciferase reporter vector containing the pig *VCL*-3′-UTR (400 bp) (0.3 μg/well) were co-transfected into the cells using Lipofectamine 3000 (Invitrogen), together with 0.1 μg/well of pRL-TK (Beyotime). At 48 h after transfection, the luciferase activity was measured with a microplate reader (TECAN) according to the manufacturer's instructions.

### Statistics

Data analysis was performed using Prism 8.0 (GraphPad). Multiple groups were compared using one-way ANOVA whereas two groups were compared with the Student *t*-test. A *p* < 0.05 was considered statistically significant.

## Results

### Characterization of SP-EVs

**Figure 1:**
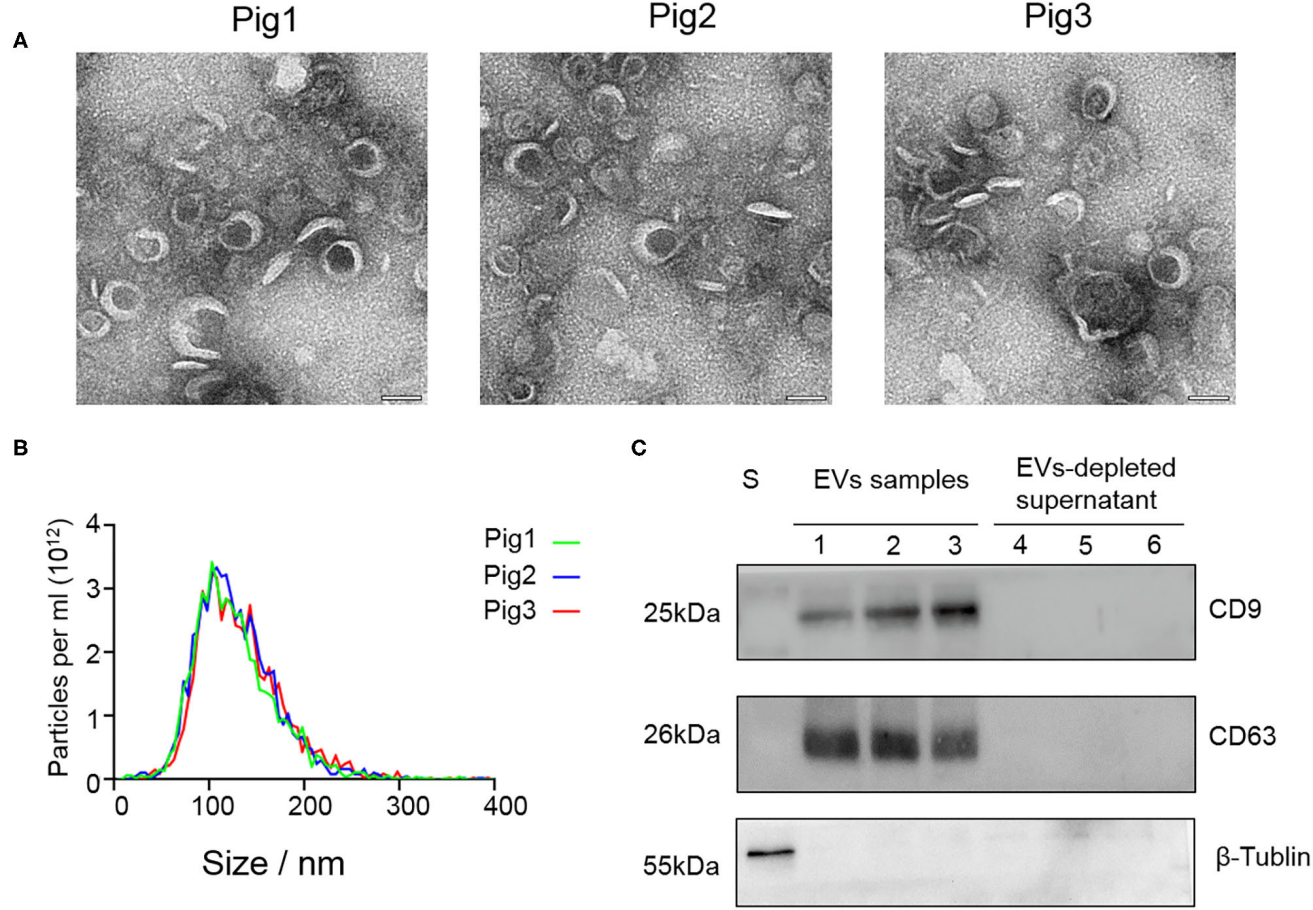
The characteristic features of EVs isolated from seminal plasma. **(A)** Transmission electron micrographs of SP-EV samples. An overview of the SP-EVs shown at a scale of 100 nm indicating the presence of a large number of EVs in boar SP-EVs. SP-EVs possess typical cup-shaped vesicle structures under the TEM. **(B)** Nanoparticle tracking analysis of particle size distribution profiles from three different SP-EV samples; most particle sizes range from 50 to 200 nm consistent with the characteristics of EVs. **(C)** Western blots of one sperm sample (S), three SP-EVs samples, and three SP-EV-depleted seminal plasma using antibodies against the EV markers CD63 and CD9.

SP-EV morphology evaluation by TEM revealed that the boar SP-EVs were presented as cup-shaped vesicle structures, which is in line with the general description of EVs (Figure 1A). NTA analysis showed that the SP-EVs collected from three different pigs had an average diameter of 121.6 ± 2.5 nm with 72.5% of the SP-EV diameter ranging from 50 to 150 nm, consistent with the documented EV size (Figure 1B). Western blotting showed that relative to the EV-depleted seminal plasma fraction, boar SP-EVs contained specific EV markers, CD9 and CD63, and have shown no cytoplasmic contaminants (β-tubulin) (Figure 1C). Taken together, these results show that the isolated boar SP-EVs indeed have all EV characteristics and that boar semen is rich in SP-EVs.

### SP-EVs Contain Several sRNA Biotypes

**Figure 2:**
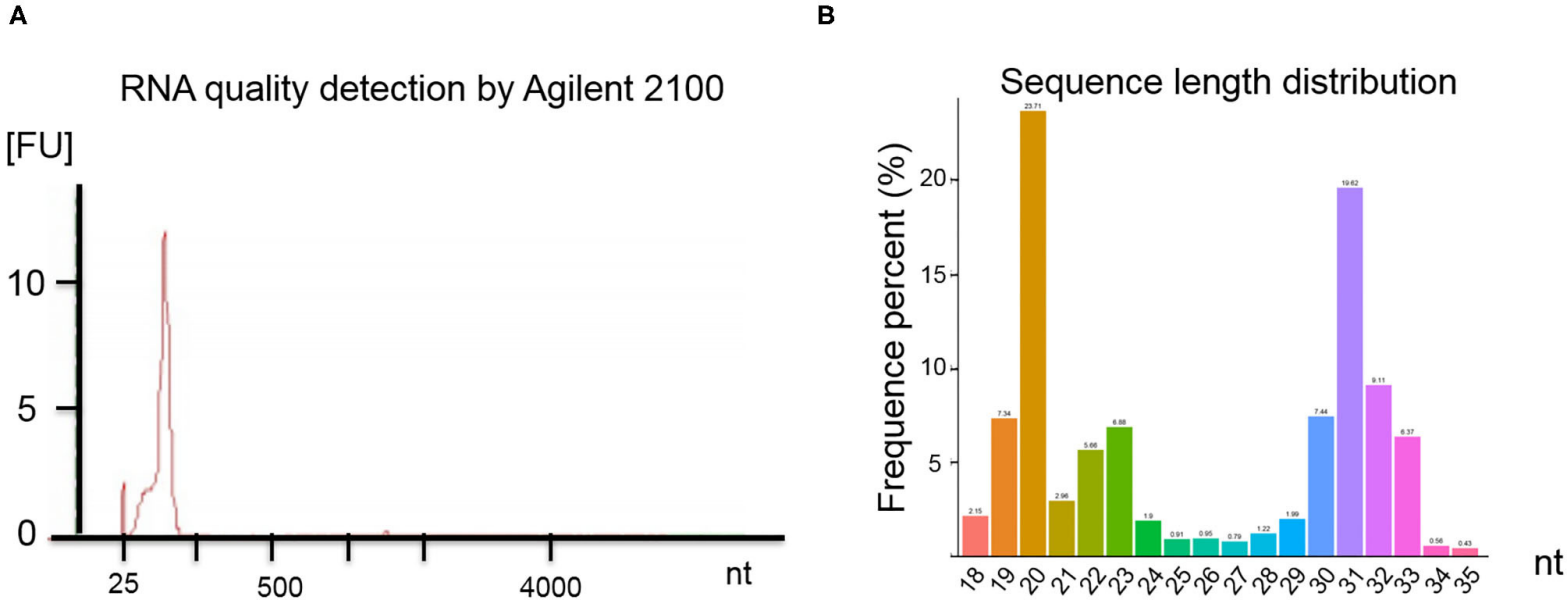
**(A)** Results of the boar SP-EV RNA quality detection by Agilent 2100. The boar SP-EVs are enriched with small RNAs. **(B)** Length distribution of RNA sequence. The different colors refer to the different base length of the identified RNAs, which have a range between 18 and 35 nt.

RNA quality was assessed by electrophoresis and Agilent 2100. Our analysis found that the SP-EV samples are enriched for sRNAs (Figure 2A) and that almost all the RNAs ranged between 18 and 35 nt in length (Figure 2B).

After exclusion of low-quality and contaminant reads, 30.11 M (94.46%) clean reads remained and were aligned to the Sus scrofa Ensemble 94 genome version (25). Mapped RNAs were sorted by biotype (Table 1); 9.45% of mapped sRNAs were identified as miRNA (including the identified novel miRNA). Unexpectedly, piRNA, which are 24–31 nt long and have important functions in germline development (14), comprised 15.25% of the library, which had not been previously reported. Protein-coding mRNA fragment constituted 25.30% of the fraction, while ribosomal RNA, tRNA, and snRNA reads accounted for 0.10, 0.01, and 0.03% of the RNA, respectively. Other sRNA fragments, including repeats, made up 17.67%, while unannotated tags represented 32.19% of total RNA. These data show that boar SP-EVs carry multiple sRNA biotypes. The sequence data have been deposited in the SRA database (SRR11870885).

**Table 1:** The sequencing reads and percentage of each RNA type in the library.

**Types**	**Total**	**Known miRNA**	**Novel miRNA**	**piRNA**	**mRNA**	**rRNA**	**tRNA**	**snRNA**	**snoRNA**	**Repeat**	**Unannotated**
Reads	19,449,493	1,740,710	96,788	2,966,589	4,920,444	19,101	1,827	4,891	840	3,437,677	6,260,626
Percent	100.00%	8.95%	0.50%	15.25%	25.30%	0.10%	0.01%	0.03%	0.00%	17.67%	32.19%

### Some Mature miRNAs Account for the Majority of the Total miRNA in Boar SP-EVs

**Figure 3:**
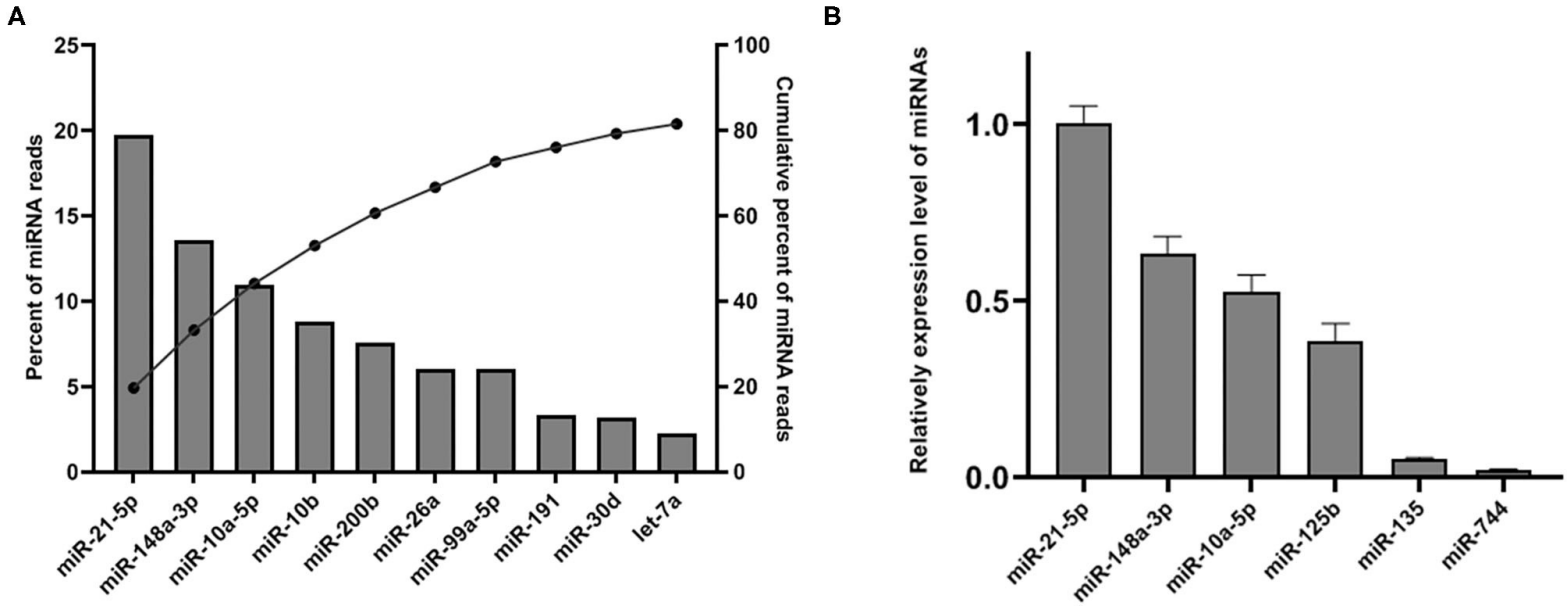
**(A)** The top 10 most abundant miRNAs in SP-EVs. Left axis and the bars: percentage of each miRNA of the total miRNA reads. Right axis and dot: cumulative percentage of miRNA reads. **(B)** qPCR analysis of miRNA expression in three additional SP-EV samples that had not been sequenced; four higher abundant and two lower abundant miRNAs (by sequencing data) were tested.

Upon mapping to miRBase release 22 (27), 288 miRNAs were identified. Although only 8.95% of the mapped sRNA in the library was identified as miRNA, we found that the top 10 abundant miRNA account for the majority of total SP-EVs miRNA reads. The most abundant miRNA is ssc-miR-21-5p, accounting for 19.72% of total SP-EVs miRNA reads. The top 5 most abundant miRNAs of the boar SP-EVs (ssc-miR-21-5p, ssc-miR-148a-3p, ssc-miR-10a-5p, ssc-miR-10b, and ssc-miR-200b) comprised 60.69% of total miRNA reads while the top 10 most abundant comprised 81.59% of total miRNA reads (Figure 3A). We validated miRNA expression levels by quantifying six selected miRNAs (four higher sequence reads miRNAs: ssc-miR-21-5p, ssc-miR-148a-3p, ssc-miR-10a-5p, and ssc-miR-125b, and two lower sequence reads miRNAs: ssc-miR-135 and ssc-miR-744) using RT-qPCR of total RNA isolated from SP-EVs from six unsequenced donors. This analysis showed that the relative expression of the six miRNAs was consistent with the RNA-seq data (Figure 3B).

### Identification of Novel miRNAs in SP-EVs

To identify potential novel miRNAs in boar SP-EVs, the unclassified tags were analyzed using miREvo (28) and mirdeep2 (29). After excluding known miRNA, rRNA, tRNA, snRNA, snoRNA, repeats, gene (protein-coding mRNA fragment), NAT-siRNA, ta-siRNA, and piRNA fragments, 37 novel mature miRNAs that had not been previously deposited in miRBase release 22 were identified (Supplementary Table 1). About 88% (85504/96788) of the novel miRNA tags started with U at the 5' end, which has been commonly observed in the 1st miRNA positions before.

### Boar SP-EVs Express High Levels of piRNAs

**Figure 4:**
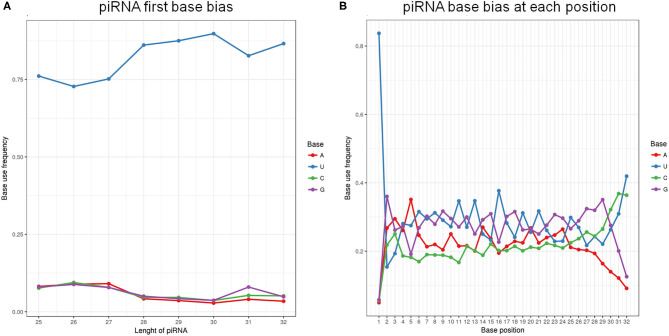
**(A)** The first base bias of different length of piRNAs. **(B)** PiRNA base bias at each position.

Analysis of piRNA profiles in the SP-EVs by NGS identified 19749 piRNAs, most of which had preference for U at position 1 and A at position 10 (Figure 4). We found that only a few kinds of the piRNAs account for the majority of total piRNAs in boar SP-EVs. The top 10 most abundant piRNAs accounted for 94.6% of the piRNA reads in the library (Table 2).

**Table 2:** The top 10 most abundant piRNAs.

**piRNA_id**	**Sequence**	**Length**	**Read count**	**TPM**
uniq_11164	GCATTGGTGGTTCAGTGGTAGAATTCTCGCC	31	2,120,736	714,873.5
uniq_11130	GCATTGGTGGTTCAGTGGTAGAATTCTCGC	30	465,605	156,949.6
uniq_56665	GCATGGGTGGTTCAGTGGTAGAATTCTCGCC	31	60,767	20,483.79
uniq_49224	GCATTTGTGGTTCAGTGGTAGAATTCTCGCC	31	50,725	17,098.76
uniq_534	TCCCTGGTGGTCTAGTGGTTAGGATTCGGC	30	30,309	10,216.78
uniq_9	TCCCTGGTGGTCTAGTGGTTAGGATT	26	23,951	8,073.582
uniq_52864	GCATTAGTGGTTCAGTGGTAGAATTCTCGCC	31	19,903	6,709.052
uniq_547	TCCCTGGTGGTCTAGTGGTTAGGATTC	27	11,984	4,039.656
uniq_461	TCCCTGGTGGTCTAGTGGTTAGGATTCGG	29	11,135	3,753.469
uniq_79645	TCCCTGGTCTAGTGGTTAGGATTCGG	26	10,523	3,547.172

### Boar SP-EVs May Have a Negative Effect on Sperm Fertility

**Figure 5:**
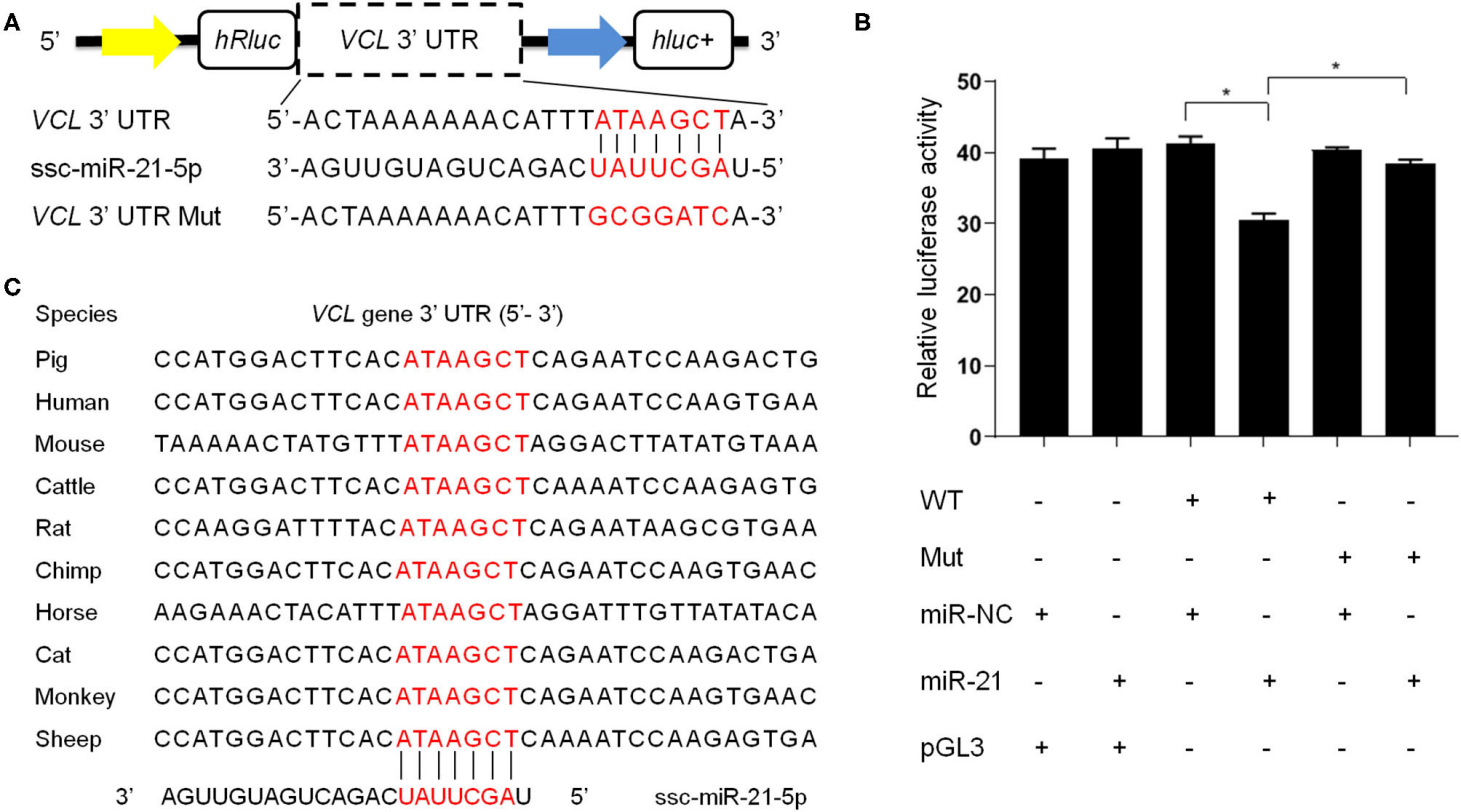
**(A)** The design of luciferase reporter. *VCL* 3' UTR sequence contains the ssc-miR-21-5p binding site; *VCL* 3' UTR Mut sequence contains mutation of the ssc-miR-21-5p binding site. **(B)** Luciferase activity was analyzed 48 h after co-transfection of PK15 cells with *VCL*3' UTR (WT) or *VCL* 3' UTR Mut plasmid (Mut) and ssc-miR-21-5p mimics (miR-21) or mimics negative control (miR-NC). pGL3 used as the basic vector of the luciferase reporter. **(C)** The ssc-miR-21-5p binding site sequences on the *VCL* 3′ UTR is conserved across species.

We then focused on some of the most abundant miRNAs in boar SP-EVs and explored their potential target genes. Among these target genes, the *VCL* gene has a score of 152 and an energy of −11.28 kcal/mol and is known to be important in sperm function (34). We then used a dual-luciferase reporter to analyze the interaction between ssc-miR-21-5p and the *VCL* gene. The luciferase reporter experiment revealed significantly suppressed luciferase activity (Figure 5). However, luciferase activity was unchanged upon co-transfection of ssc-miR-21-5p and *VCL*-3′-UTR-Mut into PK15 cells (Figure 5), indicating that ssc-miR-21-5p specifically inhibits pig *VCL* gene and may affect sperm fertility.

## Discussion and Conclusions

SP-EVs have previously been shown to be present in seminal plasma of various species, including humans (15), mouse (17), bovines (18), and boars (8, 21, 24, 35). Here, the existence of boar SP-EVs was validated by TEM, NTA, and Western blotting at the same time. EVs isolated from boar seminal plasma were shown as cup-shaped spherical structures with a mean diameter of 121.6 nm (Figures 1A,B), which are typical EV features. Western blot analysis showed that the boar SP-EVs expressed the EVs markers CD63 and CD9 and were free of cell contamination (Figure 1C). However, recent study has indicated that ultracentrifugation alone may not be sufficient to isolate clean EVs; an additional purification process such as a density gradient ultracentrifugation made with iodixanol might be applied to remove the aggregates (36). Although we tried to isolate boar SP-EVs using membrane-based affinity binding and precipitation, methods that are commonly used to isolate EVs from bioliquids, the yield was too small for TEM analysis (data are not shown).

EVs are secreted from cells and are ubiquitous in body fluids, including blood, urine, and milk. Because they can transport cargoes to distant sites (37), EVs may modulate intercellular communication (38, 39). Identifying RNAs in SP-EVs may help elucidate SP-EV function. Although miRNAs have been reported to be associated with reproduction in boars, no systematic studies have profiled sRNA boar SP-EVs. Here, analysis of SP-EV total RNA revealed that almost all of the sRNAs in boar SP-EVs were <100 nt long (Figure 2, Table 1). Of these sRNAs, miRNA and piRNA are most likely to have functional activity and were therefore thoroughly analyzed.

Our analysis found miRNAs to comprise 9.45% of total reads in SP-EVs. However, only a few miRNAs accounted for the majority of total RNAs (Figure 3). EVs that play important roles in intercellular communication have been widely accepted, and there is a study that proved that EVs could infiltrate the sperm membrane in boar (8). However, further studies should be taken to evaluate the interaction between SP-EVs and sperms. We also analyzed other abundant miRNAs. For example, DNA methyltransferase 3 beta (*DNMT3B*) gene as a candidate target of ssc-miR-148a-3p has been reported to participate in sperm development (40–42). These observations led us to hypothesize that boar SP-EVs may participate in porcine spermatogenesis. Ssc-miR-10a-5p is highly expressed in spermatogonia (43) and is also abundant in the SP-EVs, suggesting that boar SP-EVs may partially derive from testis and influence spermatogenesis. The miRNA ssc-miR-200b has been shown to target porcine spermatogenesis-associated serine-rich 2-like (*SPATS2L*) gene, which significantly affects litter size (44), suggesting that boar SP-EVs may affect sperm function and influence embryo development. Taken together, these results suggest that boar SP-EVs modulate male reproductive physiology. Further studies are required to illustrate the biological functions of boar SP-EVs. Furthermore, a recent study profiled the RNA of the boar sperm (45); it is interesting that the miR-10b, miR-191, miR-30d, and let-7a are both abundant within the sperm and SP-EVs, and they may affect sperm fertility. However, to achieve more rigorous and scientific results, we need to sequence the boar sperm and SP-EVs at the same time with a suitable sample size.

Additionally, the top 10 most abundant miRNAs may participate in other physiologic processes. For example, ssc-miR-21-5p directly impacts TLR4 signaling, while ssc-miR-21-5p promotes IL-10 production by regulating *PDCD4* expression (46) and suppressing IL-12 p35 protein by targeting *IL-12A* (47). Ssc-miR-148a-3p targets the *IL-20RB* gene to modulate immune-related function (48, 49). Let-7a miRNA belongs to the top 10 most abundant miRNAs in boar SP-EVs, and its homologs let-7c, let-7f, let-7g, and let-7i are also abundant in boar SP-EVs. These members of the Let-7 family modulate *IL-6, IL-10*, and *IL-13*, which are associated with inflammatory responses (50). These findings mean that some of the most abundant miRNAs in boar SP-EVs may have immune functions, which is in agreement with results from human SP-EVs (15). Target genes of the miRNAs listed here were summarized in Supplementary Table 2. However, how miRNAs are delivered to SP-EVs and how they regulate mRNAs warrant further study.

We have also identified piRNAs consisting of 24–31 nt in boar SP-EVs. PiRNAs are primarily expressed in germline cells (51). Here, we identified 29 SP-EV piRNAs with more than 1,000 reads, suggesting that some SP-EVs may be derived from spermatozoa or there may be a strong interaction between boar SP-EVs and spermatozoa in the semen. The source and mechanism of piRNA activity in the male genital tract have not been fully elucidated. Future studies should explore the function of these piRNAs in sperm physical function.

In conclusion, we have employed EV isolation methods to look into sRNA profiles. Our findings may further illustrate the role of miRNAs and piRNAs in sperm maturation, capacitation, acrosome reaction, and fertility, and may contribute to the development of novel therapeutic strategies for male infertility. On the other hand, the present study has limitations, such as sequencing using pooled samples to characterize the composition of EVs by ultracentrifugation alone; in addition, we could not analyze the predicted functionality of piRNAs because of the scarcity of bioinformatics tool designed for that purpose.

## Data Availability Statement

The datasets presented in this study can be found in online repositories. The names of the repository/repositories and accession number(s) can be found in the NCBI SRA database, accession SRR11870885.

## Ethics Statement

The animal study was reviewed and approved by Institutional animal care and use committee (IACUC) of South China Agricultural University. Written informed consent was obtained from the owners for the participation of their animals in this study.

## Author Contributions

ZW and LH conceived and designed the experiments. ZhiX performed the experiments, analyzed the data, and wrote the paper. YX, CZ, and QH participated in the collection of semen samples. TG, JY, EZ, SH, and ZheX participated in the implementation of the study. GC and DL critically revised the manuscript. All authors have read and approved the final version of the manuscript.

## Conflict of Interest

The authors declare that the research was conducted in the absence of any commercial or financial relationships that could be construed as a potential conflict of interest.
