# Supplementary material for: Expression Pattern of Seminal Plasma Extracellular Vesicle Small RNAs in Boar Semen
Source: Front Vet Sci. 2020 Nov 11;7:585276. doi: 10.3389/fvets.2020.585276 (PMC7685987; doi:10.3389/fvets.2020.585276)
Supplement: Supplementary file 2 [file Table_2.docx]

Supplementary Table 2: Target genes of the miRNAs listed in the Discussion

| miRNA_id | sequence | length | Target genes |
| --- | --- | --- | --- |
| ssc-miR-21-5p | UAGCUUAUCAGACUGAUGUUGA | 22 | *PDCD4, IL-12A* |
| ssc-miR-148a-3p | UCAGUGCACUACAGAACUUUGU | 22 | *IL-20RB, DNMT3B* |
| ssc-miR-200b | UAAUACUGCCUGGUAAUGAUGAC | 23 | *SPATS2L* |
| ssc-let-7a | UGAGGUAGUAGGUUGUAUAGUU | 22 | *IL-6, IL-10, IL-13* |
