# Supplementary material for: Expression Pattern of Seminal Plasma Extracellular Vesicle Small RNAs in Boar Semen
Source: Front Vet Sci. 2020 Nov 11;7:585276. doi: 10.3389/fvets.2020.585276 (PMC7685987; doi:10.3389/fvets.2020.585276)
Supplement: Supplementary file 3 [file Data_Sheet_1.docx]

Supplementary Data Sheet 1: the normal and mutant sequences of the 3’UTR (untranslated region) of the pig *VCL* gene used in this experiment

1, The normal sequences of the 3’UTR (untranslated region) of the pig *VCL* gene

TCTAGACCTCAGCATCTTTGTATAATTGCTTACCTGATATAAATGCAATATTAATGCCTTTAAAGTATGAATCTATGCCAAAGATCACCTTTTGTTTTACTAAAGATTACTTAGAGGAAAAAAGAAAAATCATGTTTGCTCTCCAAGTTCTTCCAGTGTTTTGAGACACTGGCTTACACTTTACGCCAATGTGCTTTTCTCTAATATAGTGCTCAAGACACAGTGAAGCAAATTAAAAAAGAAAAAAAAAAATCCCCGAATGCTGATTAGCGACATCACCACTAAAAAAACATTTATAAGCTAGGATTTGTTATATGCAAATATTTTCCGCCTCTTCTTTTGTTCTGTTTAAAAAAATAAAGTACATTTGTATAAGTAAAAAAAAAAAAAAAAAAAAAAAAAAAAATCTAGA

2, The mutant sequences of the 3’UTR (untranslated region) of the pig *VCL* gene

TCTAGACCTCAGCATCTTTGTATAATTGCTTACCTGATATAAATGCAATATTAATGCCTTTAAAGTATGAATCTATGCCAAAGATCACCTTTTGTTTTACTAAAGATTACTTAGAGGAAAAAAGAAAAATCATGTTTGCTCTCCAAGTTCTTCCAGTGTTTTGAGACACTGGCTTACACTTTACGCCAATGTGCTTTTCTCTAATATAGTGCTCAAGACACAGTGAAGCAAATTAAAAAAGAAAAAAAAAAATCCCCGAATGCTGATTAGCGACATCACCACTAAAAAAACATTTGCGGATCAGGATTTGTTATATGCAAATATTTTCCGCCTCTTCTTTTGTTCTGTTTAAAAAAATAAAGTACATTTGTATAAGTAAAAAAAAAAAAAAAAAAAAAAAAAAAAATCTAGA
